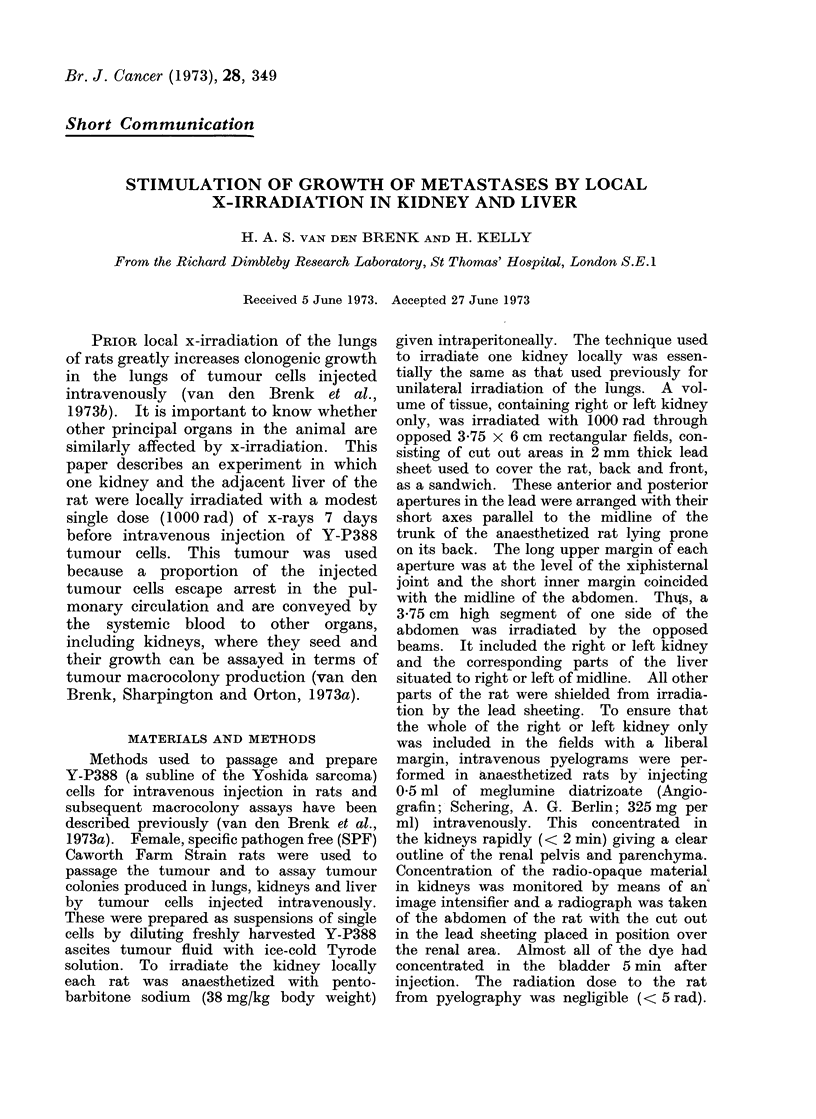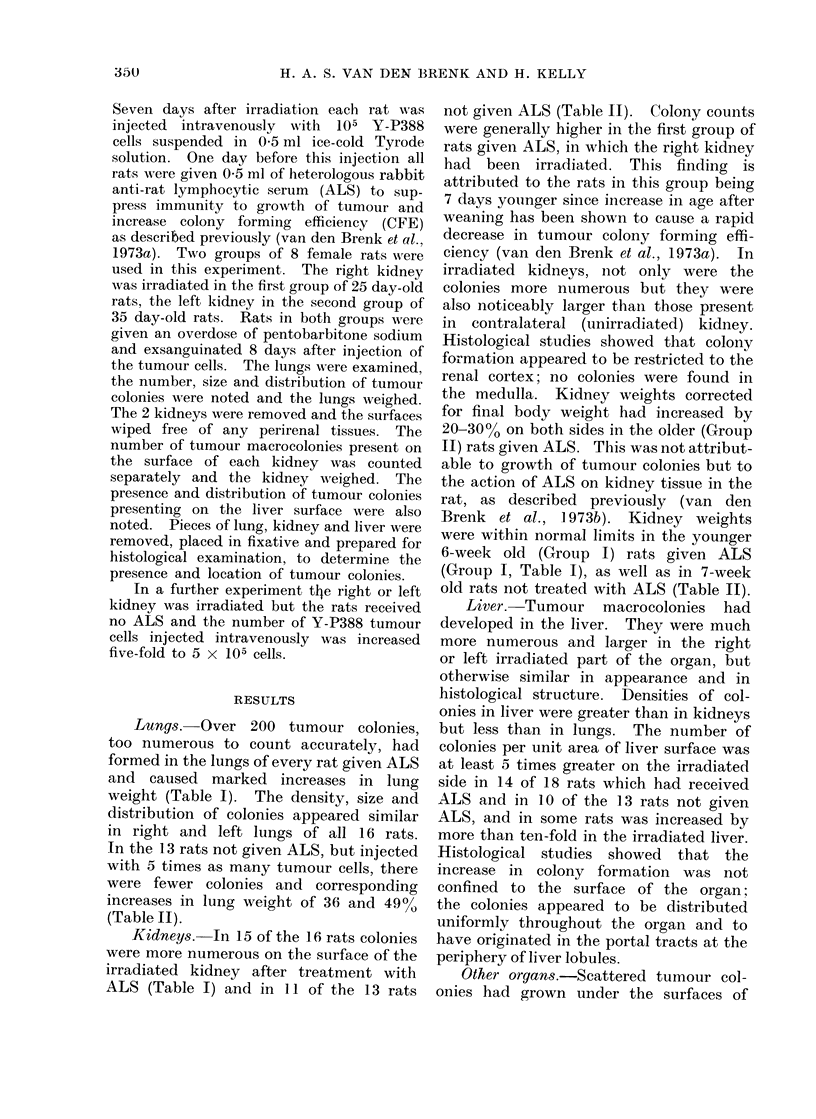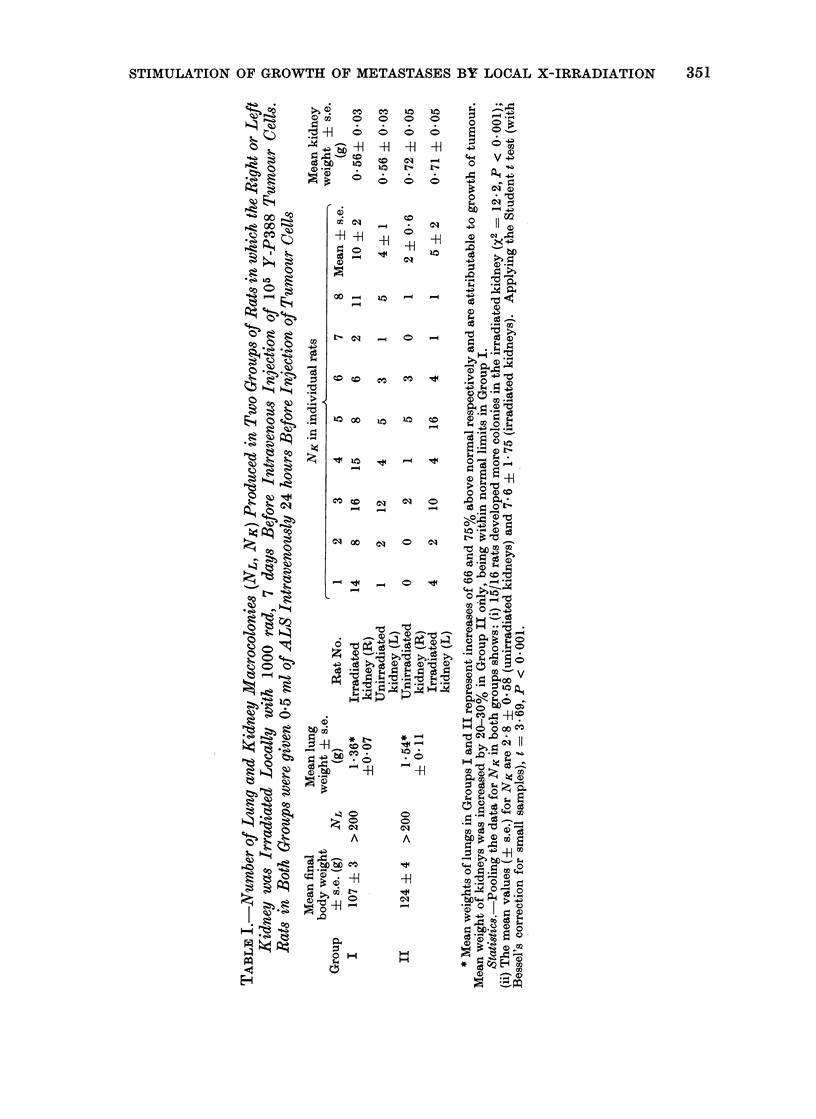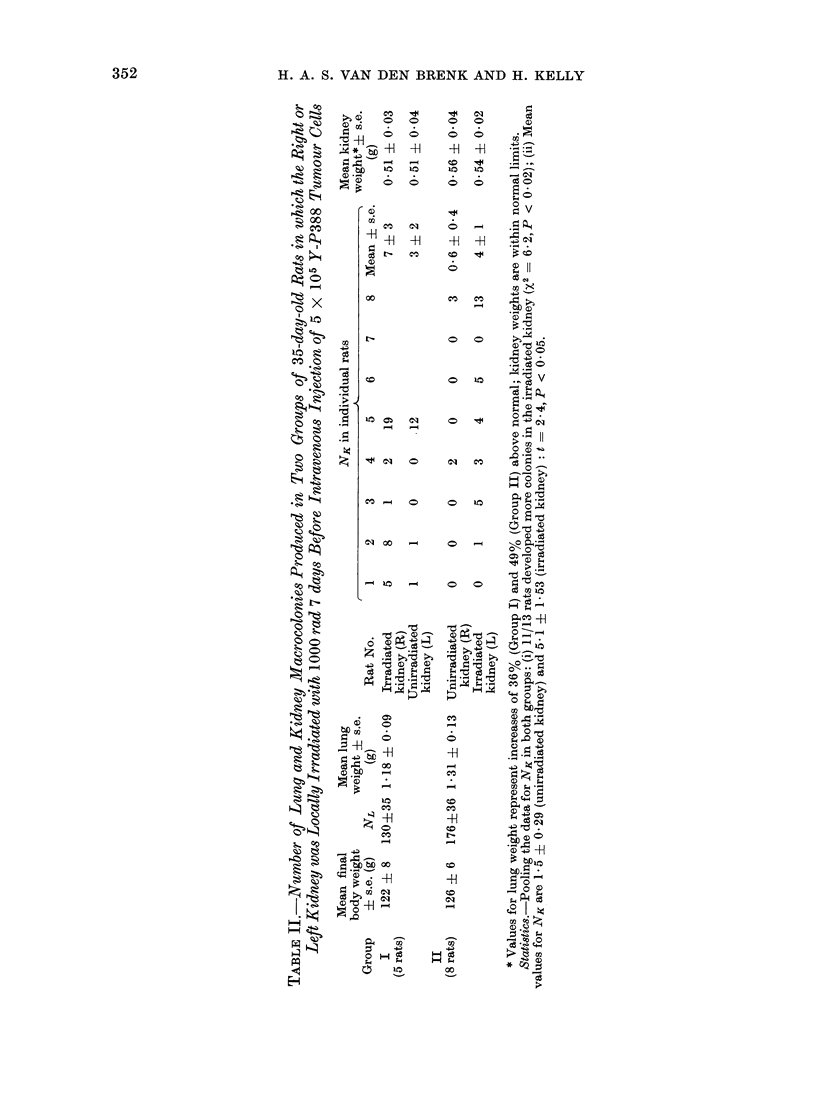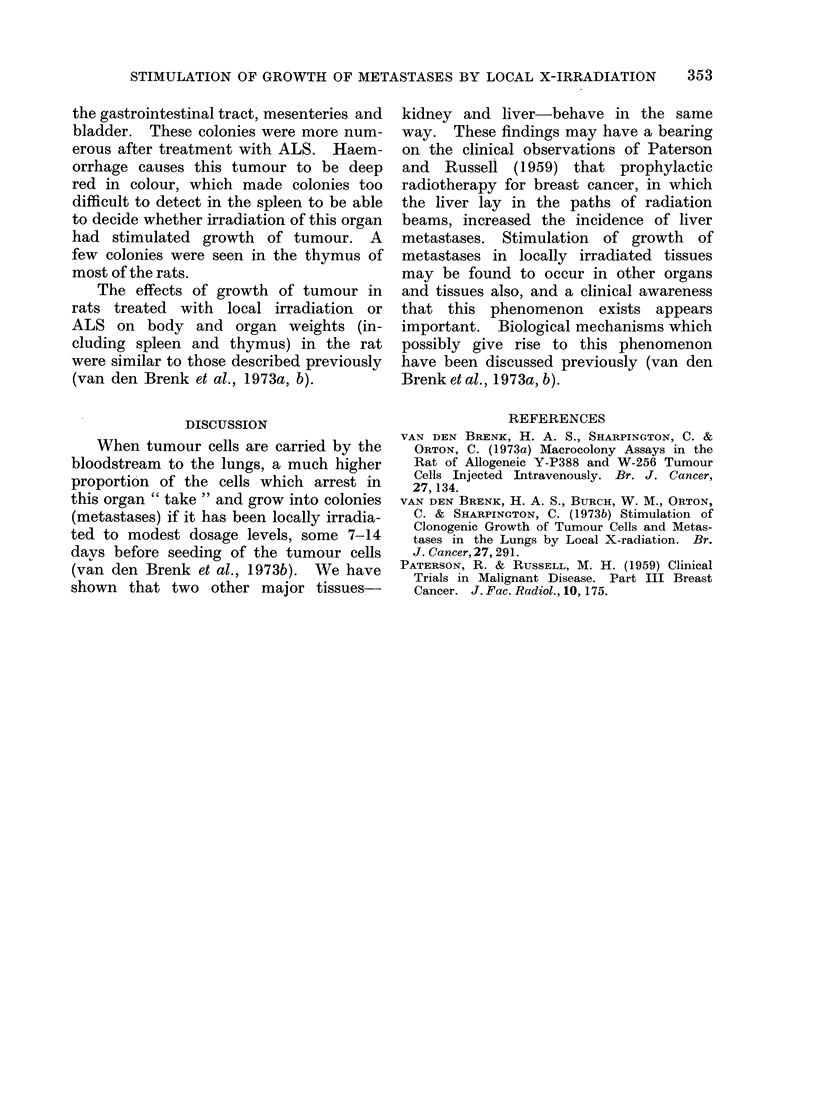# Stimulation of growth of metastases by local x-irradiation in kidney and liver.

**DOI:** 10.1038/bjc.1973.158

**Published:** 1973-10

**Authors:** H. A. Van Den Brenk, H. Kelly


*Br. J. Cancer* (1973), **28**, 349

***Short Communication***

### STIMULATION OF GROWTH OF METASTASES BY LOCAL X-IRRADIATION IN KIDNEY AND LIVER

H. A. S. VAN DEN BRENK AND H. KELLY

*From the Richard Dimbleby Research Laboratory, St Thomas' Hospital, London S.E.*1

Received 5 June 1973. Accepted 27 June 1973

PRIOR local x-irradiation of the lungs of rats greatly increases clonogenic growth in the lungs of tumour cells injected intravenously (van den Brenk *et al.*, 1973*b*). It is important to know whether other principal organs in the animal are similarly affected by x-irradiation. This paper describes an experiment in which one kidney and the adjacent liver of the rat were locally irradiated with a modest single dose (1000 rad) of x-rays 7 days before intravenous injection of Y-P388 tumour cells. This tumour was used because a proportion of the injected tumour cells escape arrest in the pulmonary circulation and are conveyed by the systemic blood to other organs, including kidneys, where they seed and their growth can be assayed in terms of tumour macrocolony production (van den Brenk, Sharpington and Orton, 1973*a*).

### MATERIALS AND METHODS

Methods used to passage and prepare Y-P388 (a subline of the Yoshida sarcoma) cells for intravenous injection in rats and subsequent macrocolony assays have been described previously (van den Brenk *et al.*, 1973*a*). Female, specific pathogen free (SPF) Caworth Farm Strain rats were used to passage the tumour and to assay tumour colonies produced in lungs, kidneys and liver by tumour cells injected intravenously. These were prepared as suspensions of single cells by diluting freshly harvested Y-P388 ascites tumour fluid with ice-cold Tyrode solution. To irradiate the kidney locally each rat was anaesthetized with pentobarbitone sodium (38 mg/kg body weight) given intraperitoneally. The technique used to irradiate one kidney locally was essentially the same as that used previously for unilateral irradiation of the lungs. A volume of tissue, containing right or left kidney only, was irradiated with 1000 rad through opposed 3·75 × 6 cm rectangular fields, consisting of cut out areas in 2 mm thick lead sheet used to cover the rat, back and front, as a sandwich. These anterior and posterior apertures in the lead were arranged with their short axes parallel to the midline of the trunk of the anaesthetized rat lying prone on its back. The long upper margin of each aperture was at the level of the xiphisternal joint and the short inner margin coincided with the midline of the abdomen. Thus, a 3·75 cm high segment of one side of the abdomen was irradiated by the opposed beams. It included the right or left kidney and the corresponding parts of the liver situated to right or left of midline. All other parts of the rat were shielded from irradiation by the lead sheeting. To ensure that the whole of the right or left kidney only was included in the fields with a liberal margin, intravenous pyelograms were performed in anaesthetized rats by injecting 0·5 ml of meglumine diatrizoate (Angiografin; Schering, A. G. Berlin; 325 mg per ml) intravenously. This concentrated in the kidneys rapidly ($<$ 2 min) giving a clear outline of the renal pelvis and parenchyma. Concentration of the radio-opaque material in kidneys was monitored by means of an image intensifier and a radiograph was taken of the abdomen of the rat with the cut out in the lead sheeting placed in position over the renal area. Almost all of the dye had concentrated in the bladder 5 min after injection. The radiation dose to the rat from pyelography was negligible ($<$ 5 rad).

Seven days after irradiation each rat was injected intravenously with $10^5$ Y-P388 cells suspended in 0·5 ml ice-cold Tyrode solution. One day before this injection all rats were given 0·5 ml of heterologous rabbit anti-rat lymphocytic serum (ALS) to suppress immunity to growth of tumour and increase colony forming efficiency (CFE) as described previously (van den Brenk *et al.*, 1973*a*). Two groups of 8 female rats were used in this experiment. The right kidney was irradiated in the first group of 25 day-old rats, the left kidney in the second group of 35 day-old rats. Rats in both groups were given an overdose of pentobarbitone sodium and exsanguinated 8 days after injection of the tumour cells. The lungs were examined, the number, size and distribution of tumour colonies were noted and the lungs weighed. The 2 kidneys were removed and the surfaces wiped free of any perirenal tissues. The number of tumour macrocolonies present on the surface of each kidney was counted separately and the kidney weighed. The presence and distribution of tumour colonies presenting on the liver surface were also noted. Pieces of lung, kidney and liver were removed, placed in fixative and prepared for histological examination, to determine the presence and location of tumour colonies.

In a further experiment the right or left kidney was irradiated but the rats received no ALS and the number of Y-P388 tumour cells injected intravenously was increased five-fold to $5 \times 10^5$ cells.

### RESULTS

*Lungs.*—Over 200 tumour colonies, too numerous to count accurately, had formed in the lungs of every rat given ALS and caused marked increases in lung weight (Table I). The density, size and distribution of colonies appeared similar in right and left lungs of all 16 rats. In the 13 rats not given ALS, but injected with 5 times as many tumour cells, there were fewer colonies and corresponding increases in lung weight of 36 and 49% (Table II).

*Kidneys.*—In 15 of the 16 rats colonies were more numerous on the surface of the irradiated kidney after treatment with ALS (Table I) and in 11 of the 13 rats not given ALS (Table II). Colony counts were generally higher in the first group of rats given ALS, in which the right kidney had been irradiated. This finding is attributed to the rats in this group being 7 days younger since increase in age after weaning has been shown to cause a rapid decrease in tumour colony forming efficiency (van den Brenk *et al.*, 1973*a*). In irradiated kidneys, not only were the colonies more numerous but they were also noticeably larger than those present in contralateral (unirradiated) kidney. Histological studies showed that colony formation appeared to be restricted to the renal cortex; no colonies were found in the medulla. Kidney weights corrected for final body weight had increased by 20–30% on both sides in the older (Group II) rats given ALS. This was not attributable to growth of tumour colonies but to the action of ALS on kidney tissue in the rat, as described previously (van den Brenk *et al.*, 1973*b*). Kidney weights were within normal limits in the younger 6-week old (Group I) rats given ALS (Group I, Table I), as well as in 7-week old rats not treated with ALS (Table II).

*Liver.*—Tumour macrocolonies had developed in the liver. They were much more numerous and larger in the right or left irradiated part of the organ, but otherwise similar in appearance and in histological structure. Densities of colonies in liver were greater than in kidneys but less than in lungs. The number of colonies per unit area of liver surface was at least 5 times greater on the irradiated side in 14 of 18 rats which had received ALS and in 10 of the 13 rats not given ALS, and in some rats was increased by more than ten-fold in the irradiated liver. Histological studies showed that the increase in colony formation was not confined to the surface of the organ; the colonies appeared to be distributed uniformly throughout the organ and to have originated in the portal tracts at the periphery of liver lobules.

*Other organs.*—Scattered tumour colonies had grown under the surfaces of the gastrointestinal tract, mesenteries and bladder. These colonies were more numerous after treatment with ALS. Haemorrhage causes this tumour to be deep red in colour, which made colonies too difficult to detect in the spleen to be able to decide whether irradiation of this organ had stimulated growth of tumour. A few colonies were seen in the thymus of most of the rats.

The effects of growth of tumour in rats treated with local irradiation or ALS on body and organ weights (including spleen and thymus) in the rat were similar to those described previously (van den Brenk *et al.*, 1973*a*, *b*).

### DISCUSSION

When tumour cells are carried by the bloodstream to the lungs, a much higher proportion of the cells which arrest in this organ "take" and grow into colonies (metastases) if it has been locally irradiated to modest dosage levels, some 7–14 days before seeding of the tumour cells (van den Brenk *et al.*, 1973*b*). We have shown that two other major tissues—kidney and liver—behave in the same way. These findings may have a bearing on the clinical observations of Paterson and Russell (1959) that prophylactic radiotherapy for breast cancer, in which the liver lay in the paths of radiation beams, increased the incidence of liver metastases. Stimulation of growth of metastases in locally irradiated tissues may be found to occur in other organs and tissues also, and a clinical awareness that this phenomenon exists appears important. Biological mechanisms which possibly give rise to this phenomenon have been discussed previously (van den Brenk *et al.*, 1973*a*, *b*).

### REFERENCES

van den Brenk, H. A. S., Sharpington, C. & Orton, C. (1973*a*) Macrocolony Assays in the Rat of Allogeneic Y-P388 and W-256 Tumour Cells Injected Intravenously. *Br. J. Cancer*, **27**, 134.

van den Brenk, H. A. S., Burch, W. M., Orton, C. & Sharpington, C. (1973*b*) Stimulation of Clonogenic Growth of Tumour Cells and Metastases in the Lungs by Local X-radiation. *Br. J. Cancer*, **27**, 291.

Paterson, R. & Russell, M. H. (1959) Clinical Trials in Malignant Disease. Part III Breast Cancer. *J. Fac. Radiol.*, **10**, 175.